# Key Findings and Lessons from an Evaluation of the Rockefeller Foundation's Disease Surveillance Networks Initiative

**DOI:** 10.3402/ehtj.v6i0.19959

**Published:** 2013-01-25

**Authors:** Nancy MacPherson, Ann Marie Kimball, Charlanne Burke, Neil Abernethy, Sandra Tempongko, Jakob Zinsstag

**Affiliations:** 1 Rockefeller Foundation, United States; 2 School of Public Health, University of Washington, United States; 3 Rockefeller Foundation, United States; 4 Department of Medical Education and Biomedical Informatics, University of Washington, United States; 5 Southeast Asian Ministers of Education Tropical Medicine and Public Health Network, Thailand; 6 Swiss Tropical and Public Health Institute, Switzerland

The Rockefeller Foundation has a long history of providing support for disease surveillance in Asia, globally, and more recently in Africa (1–3). Most recently, from 2007–2012, the Foundation provided $22 million in support for the Disease Surveillance Networks (DSN) Initiative with the goal of contributing to the mitigation of disease outbreaks by supporting transnational and inter-disciplinary networks aimed at strengthening national, regional, and global disease surveillance and response systems. Specifically, the DSN Initiative aimed to build individual and institutional capacity to conduct disease surveillance and response efficiently and effectively; build bridges between disease surveillance networks and international agencies to increase the effectiveness of global response systems; and strengthen connections between animal health, human health, and environmental health through a “One Health” approach.

The underlying hypothesis of the Initiative was that robust trans-boundary, multi-sectoral and cross-disciplinary collaborative networks lead to improved disease surveillance and response. In 2009–2010, the Rockefeller Foundation undertook an independent evaluation of the Initiative, with a focus on the Mekong Region in Asia, Eastern and Southern Africa, and with global partners involved in the Initiative. Evaluation teams led by the Southeast Asian Ministers of Education Tropical Medicine and Public Health Network (SEAMEO TropMed), the Swiss Tropical and Public Health Institute and African Public Health Research Centre, and the University of Washington assessed the validity of the underlying hypothesis and sought evidence of achievements, challenges, and lessons related to DSN formation and sustainability, capacity, tools, and transdisciplinary approaches (Text Box 1). Evaluation teams collected different types of data: (i) through field visits and interviews with key stakeholders; (ii) from a detailed analysis of Foundation portfolio grants; and (iii) through an innovative network analysis of the growth and connectivity of regional and global networks.

***Text Box 1*. Intended outcomes of the Rockefeller Foundation support to the Disease Surveillance Networks (DSN) Initiative**

***Outcome 1: Networks:*** Trans-boundary disease surveillance networks in Southeast Asia, and in Eastern and Southern Africa are formed, sustained, and evolve in order to enable disease surveillance practitioners to collaborate, share information, and learn how to more effectively address disease threats.

***Outcome 2: Capacity:*** Disease surveillance practitioners and their institutions strengthen, apply, and distribute technical and communication skills in disease surveillance to more effectively address disease threats.

***Outcome 3: Tools:*** Disease surveillance practitioners have access to – and use –improved tools and methods to effectively and efficiently monitor, share and report information; and respond to disease threats.

***Outcome 4: Transdisciplinary Leadership in One Health:*** Policy makers, human health and veterinary practitioners take a trans-disciplinary approach to policy and practice in animal and human health while emphasizing “One Health” principles at global, regional and local levels.

In commissioning the independent evaluation, the Foundation was interested in knowing whether the Foundation's work in disease surveillance was relevant to current global and regional trends and challenges in disease surveillance; whether it was efficiently and effectively used to improve skills and capacity for early detection and response; whether it was helpful to containing infectious disease outbreaks, thereby saving lives and livelihoods; and whether it is sustainable in the longer term.

This article discusses key findings of the evaluation and implications for those involved in strengthening the field of disease surveillance. The evaluation represents the only formal set of evaluations conducted thus far on any of the Connecting Organizations for Regional Disease Surveillance (CORDS) networks.

## Evidence of Achievements

Evidence collected during the evaluation indicates that the Initiative made great progress towards achieving its intended outcomes. Very broadly, with its partners, the Initiative provided vision and support that helped to establish new fields of practice in One Health, as well as global health diplomacy; built substantial capacity through targeted high quality grantee support; and fostered trust among key stakeholders. These efforts were facilitated by the generation of new knowledge in the application of networking to promote global health and in the governance of sub-regional networks; and by the elaboration and adoption of innovative Information Technology (IT) tools for surveillance and response. Specific findings are described below.

### Effectiveness and Sustainability of the Networks (Intended Outcome 1)

The evaluators found conclusive and compelling evidence that effective and sustainable disease surveillance networks have been well established in Asia (notably the Mekong Basin Disease Surveillance [MBDS] network) (4) and that promising networks are emerging in Africa (5–6). Field visit and interview data indicate that networks supported by the Rockefeller Foundation DSN Initiative work have demonstrated effectiveness in reporting and containing outbreaks such as dengue, severe acute respiratory syndrome (SARS), and influenza. Stakeholders on every level validated the relevance and utility of the networked approach to disease surveillance. The regional network structure is seen as one that promotes the sharing of knowledge, resources, and best practices and thereby improves country-level efficiency in adopting effective surveillance and response systems. Network structures are also seen as a way of distributing capacity and assuring timely access to technical capacity in resource poor settings. Finally, they are seen as a way to build deeper and more extensive global, regional, and local ties between disease surveillance organizations and countries. These ties not only increase access to knowledge sharing, but they also create new pathways for the flow of critical information.

Major factors driving the success of regional DSNs in Asia and Africa include: trust; transparency; a cooperative spirit; and partners with a sustained vision of a strong informal network. Government ownership, leadership and political will are also essential for success.

**Fig. 1:**
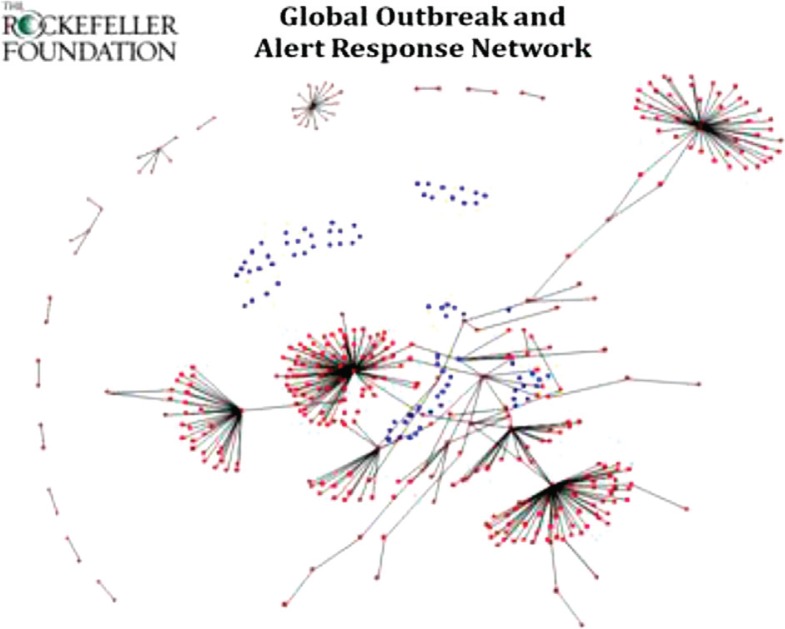
Disease surveillance organizations before involvement in the Disease Surveillance Network (DSN) Initiative. Blue dots represent DSN grantees from Asia and Africa when they were first supported by the Initiative. Source: Rockefeller Foundation and Neil Abernethy.

**Fig. 2:**
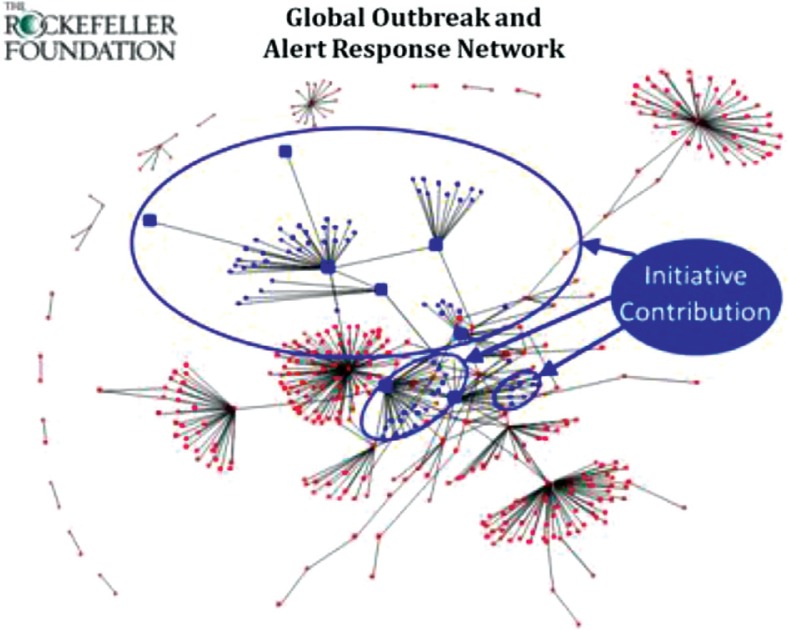
Grantees after involvement with the DSN Initiative – better connected locally, regionally and globally. Connections circled in blue are those that formed after the DSN Initiative began supporting regional DSNs in Asia and Africa. Source: Rockefeller Foundation and Neil Abernethy.

As shown in Figs. 1 and 2, a systematic network mapping analysis of the DSN Initiative presence in the World Health Organization (WHO)'s Global Outbreak and Alert Response Network (GOARN) shows that a small number of DSN grantees act as hubs in the larger network, connecting dozens of organizations to the global disease surveillance community and forming additional connections between organizations and countries already represented. In addition, many countries with minimal network representation benefit from organizational ties with DSN grantees. Fig. 1 shows individual disease surveillance grantees of the Rockefeller Foundation DSN Initiative prior to their involvement with the Initiative, with grantees not being well connected either to GOARN or to existing regional networks. Fig. 2 shows the growing connectedness between Initiative grantees and other organizations and countries. With Initiative support, grantees are connected both geographically and thematically with key players in disease surveillance at the global, regional, and local levels.

**Fig. 3:**
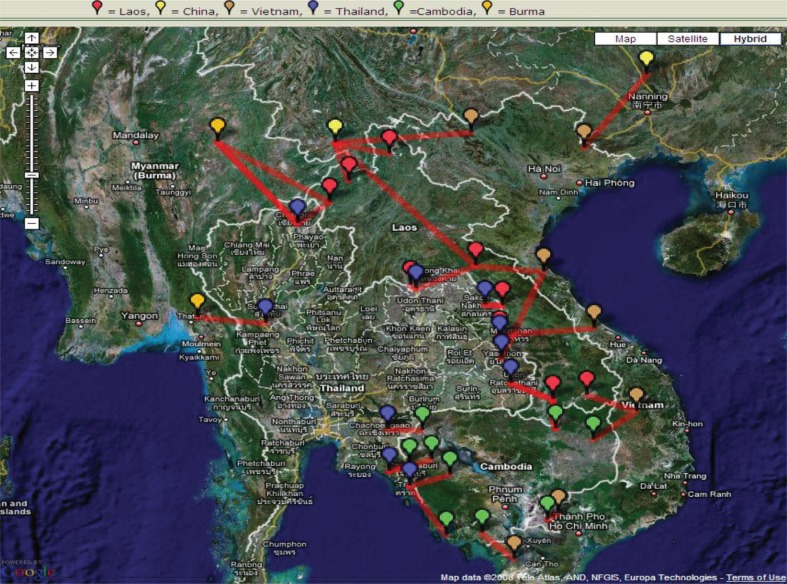
Cross border surveillance sites in the six countries of the Mekong Basin Disease Surveillance Network (MBDS): Thailand, Cambodia, Lao PDR, Vietnam, Myanmar and Yunnan Province, China. Source: Rockefeller Foundation.

Not only has connectedness increased, but also critical networks at local and regional levels have become stronger as a result of the DSN Initiative. Border crossings are considered the most critical areas for containing the spread of highly infectious diseases and pandemics from country to country. Yet, they are often the most isolated and with least capacity in the whole disease surveillance system. As shown in Fig. 3, from 2004 to 2010, local cross-border surveillance sites in the six countries of the Mekong Basin Disease Surveillance (MBDS) network increased from four to 24, covering most of the critical trade routes (4).

### New Capacity (Intended Outcome 2)

A second intended outcome of the DSN Initiative was to build capacity to more effectively address infectious disease threats. Portfolio, field visit, and interview data indicate that the Initiative supported activities that contributed positively and substantively to building individual, institutional, and network capacity in epidemiology, surveillance, and outbreak investigation and response. Specifically, the Initiative supported the provision and, with local partners, development of training, thought leadership, curricula, tools, technical support, and forums for learning, sharing, and developing knowledge and best practices. Additionally, Initiative support has facilitated substantial interaction not only among different networks, but also among different sectors (e.g., animal and human health, livestock, agriculture, transportation, security, immigration). For example, MBDS is cited by network participants, peers, and partners as being fundamental in promoting the sharing of knowledge, resources, and best practices to improve the Mekong countries’ surveillance and response systems. As another example, the East Africa Integrated Disease Surveillance Network (EAIDSNet) and Southern African Centre for Infectious Disease Surveillance (SACIDS) are jointly testing mobile phone surveillance tools (See Text Box 2 in [6]).

### New Tools (Intended Outcome 3)

Grantees of the DSN Initiative asked for, helped to develop, and piloted a wide range of new and innovative tools for real time transmission of clinical observations and expert advice using mobile technologies, Geographical Information Systems (GIS), and web-based platforms. While most tools were well received and proved to be useful, some need more time for integration because of varying technical and cultural practices.

### Transdisciplinary Leadership in One Health (Intended Outcome 4)

The inclusion of One Health as a central focus of the Initiative was perceived to be highly relevant to the challenges in the field of disease surveillance, especially given that the animal and human health disciplines have operated in silos for decades. The One Health concept is now widely known by local, regional, and global practitioners and has received funding from other major donors (e.g., U.S. agencies, the Asia Pacific Emerging Disease Framework [APSED] and other WHO regional frameworks) as a result of extensive efforts by Initiative partners and grantees to communicate the concept and its practical application. For example, the recent USAID Emerging Pandemic Threats Program fund of $400 million is built directly on the work of Initiative grantees and their partners (7).

### Overall Impact

The evaluators concluded that the Initiative has had significant impact on the field of disease surveillance policy and practice at both regional and global levels, particularly through its support of regional networks in Asia and through its early application of One Health principles in Africa and worldwide. In Asia, the Initiative contributed significantly to containing outbreaks in the Mekong Basin countries by supporting changes at the provincial, district, and village levels that helped to prevent the spread of several different outbreaks at different times and in different locations, saving lives and lessening the negative impact on the livelihoods of some of the world's poorest people. The Initiative has ensured its continued relevance at both regional and global levels not only by being an early adopter of the One Health perspective, but also by listening to its partners and network members, shifting its focus over time in response to changing needs and trends in human/animal health, and embracing the growing need for health diplomacy.

Initiative grantees’ policy reach extends beyond One Health. The regional disease surveillance networks supported by the Initiative and described in this supplement have become key players in developing a wide range of regional and global policies and in the collaborative implementation of those policies. For example, the Asian Development Bank modeled its Greater Mekong investments on Initiative work. Some of this reach can be credited to the Rockefeller Foundation's long history of work in the field of public health and its credibility and perceived leadership among development partners in linking theory, research, policy, and practice. Indeed, it was the Foundation's history of work in public health that served as a solid rationale for its involvement in the DSN Initiative.

### Challenges for the Rockefeller Foundation

The Evaluation highlighted several key challenges faced by the Initiative and lessons learned and revealed areas where the Foundation needs to strengthen its own systems and strategies (7–9). First, despite gains in capacity in skills and practical knowledge, there is uneven documentation in both the peer-reviewed and non peer-reviewed literature of the work of the Initiative. Second, Foundation information systems were unable to provide timely and complete information on grantee outputs. Third, efficiency and sustainability would be enhanced by coordination with other large funders and partners. For example, the simultaneous growth of networks such as WHO's Global Outreach Alert and Response Network, the Global Laboratories Directory and Network, and the U.S. Centers for Disease Control (CDC) were not planned to interoperate with the DSN grantees, limiting opportunities to efficiently work and grow together. Fourth, a formal exit strategy to guide the Foundation when concluding the work of the Initiative was not put into place early enough. Finally, monitoring and evaluation practices within grants were informal and not well documented.

## Recommendations

The evaluation teams made detailed recommendations in three main categories for the Foundation's work in Asia, Africa, and globally (7–9): (i) ensure sustainability of the substantial gains made by the Foundation's grantee partners; (ii) promote the One Health concept and practice; and (iii) capture the lessons and achievements of the Initiative and communicate these widely.

In response to these recommendations, the Foundation convened all DSN grantees in April 2011 in Nairobi, Kenya, to consolidate the experience and learning of the Initiative, disseminate evaluation findings, and consider the needs of the field going forward. The Foundation also supported the legal institutionalization of MBDS to ensure continuity of the work in Asia and provided further time-bound support to SACIDS. In addition, in partnership with the Nuclear Threat Initiative (NTI), Fondation Mérieux and the Skoll Global Threats Fund, the Foundation supported the legal institutionalization of CORDS to ensure its sustainability. Finally, the Foundation provided transition support for its One Health work, with the expectation that this will come to fruition and be highlighted at the Prince Mahidol Award Conference in Bangkok, 2013, where the theme is “A World United Against Infectious Diseases: Cross-Sectoral Solutions.”

## Conclusion

In conclusion, the evaluation of the Disease Surveillance Networks Initiative was an important undertaking within the Foundation. Lessons learned have been shared internally and are being applied to the current and future work of the Foundation. The results demonstrated the value and merit of the Foundation's investments in supporting disease surveillance networks and other grantee work in this portfolio, resulting in a successful application to the Board of Trustees of the Foundation for additional funding to support sustainability among key partners. Sustainability of the progress achieved and relationships nurtured through the DSN Initiative is important to the Foundation, especially as internal efforts are directed elsewhere. Building on the clear evidence of ongoing relevance, motivation, and sustained engagement in the networks by members and partners, and solid interest from other funders, the Foundation has supported institutional independence of MBDS and CORDS.
